# Is there a need for a technical certification system for gynecological robotic surgery? Questionnaire survey of members of the Japan Society of Gynecologic and Obstetric Endoscopy and Minimally Invasive Therapy

**DOI:** 10.1007/s11701-022-01520-8

**Published:** 2023-01-09

**Authors:** Hiroaki Komatsu, Osamu Hiraike, Rie Fukuhara, Yoshihito Yokoyama, Nobutaka Takahashi, Hirotaka Nishi, Tsukasa Baba, Takuma Fujii, Jo Kitawaki, Hiroaki Kobayashi, Masaki Mandai, Technical Certification System for Gynecological Robotic Surgery Task Force of the Japan Society of Gynecologic, Obstetric Endoscopy, Minimally Invasive Therapy

**Affiliations:** 1 Department of Obstetrics and Gynecology, Tottori University School of Medicine, Tottori, Japan; 2 Department of Obstetrics and Gynecology, Graduate School of Medicine, University of Tokyo, Tokyo, Japan; 3 Department of Obstetrics and Gynecology, Graduate School of Medicine, Hirosaki University, Hirosaki, Aomori Japan; 4 Department of Gynecology, Shizuoka Cancer Center, Sunto-Gun, Shizuoka, Japan; 5 Department of Obstetrics and Gynecology, Tokyo Medical University, Tokyo, Japan; 6 Department of Obstetrics and Gynecology, Iwate Medical University School of Medicine, Iwate, Japan; 7 Department of Obstetrics and Gynecology, School of Medicine, Fujita Health University, 1-98, Dengakugakubo, Toyoake, Aichi Japan; 8 Department of Gynecology and Obstetrics, Kyoto Prefectural University of Medicine, Kyoto, Japan; 9 Department of Gynecology and Obstetrics, Kagoshima University Graduate School of Medical and Dental Sciences, Kagoshima, Japan; 10 Department of Gynecology and Obstetrics, Kyoto University Graduate School of Medicine, Kyoto, Japan

**Keywords:** Japan, Training system, Robotic surgery, Survey, Technical certification

## Abstract

The Japan Society of Gynecologic and Obstetric Endoscopy and Minimally Invasive Therapy (JSGOE) introduced a system for the certification of laparoscopic surgeons in 2002 and a system for the certification of facilities in 2014. We examined the opinions of the members of the Japan Society of Gynecologic and Obstetric Endoscopy and Minimally Invasive Therapy (JSGOE) regarding the necessity of a certification training system and a technical certification system for robotic surgery skills in Japan. Members of the JSGOE were surveyed with two questionnaires. Overall, 870 and 519 participants responded to the first and second questionnaires, respectively. Half of the respondents indicated that both systems were necessary. The breakdown by age and qualifications showed that this was especially true for the younger generation and respondents with more experience with robotic surgery. Overall, 40% of the respondents judged that a certification system for robotic surgery alone (with or without certification in laparoscopic surgery but with a requirement of experience in laparoscopic surgery) would be necessary. The opinions of the JSGOE members on making a certification system for robotic surgery were split into two clear-cut camps. Thus, we must further seek the extent of public demand for using a public survey so that a final decision can be made on whether to establish this system.

## Introduction

 The number of robotic-assisted surgeries performed increases annually. In the USA, it was approved by the Food and Drug Administration in 2005 and today accounts for 45% of all surgeries [1]. Similarly, the number of robotic surgeries performed has been increasing in Europe, the UK, and Australia [2, 3]. However, complications are expected to increase when new technologies and systems are introduced. In fact, deaths from robotic surgery for gynecological disease have been reported in the USA [4].

The Japan Society of Gynecologic and Obstetric Endoscopy and Minimally Invasive Therapy (JSGOE) introduced a system for the certification of laparoscopic surgeons in 2002 and a system for the certification of facilities in 2014 to reduce unnecessary complications, especially when introducing new techniques, and help to maintain the public health. The number of perioperative complications associated with laparoscopic surgery has decreased [5]. This number is lower in Japan than that reported in other countries [6–8]. Thus, the JSGOE has provided safe surgeries to the public and has increased its centripetal force by formulating a technical certification system and certified training facilities and establishing a nationwide system for registering complications. Although robotic surgery reportedly has a shorter operation time and the surgical technique is more easily stabilized than laparoscopic surgery, there is debate in Japan on whether a similar institutional design as that for laparoscopic surgery is necessary for robotic surgery [9, 10].

 This study aimed to determine the necessity for and the concrete requirements of a certification training system and a technical certification system for robotic surgery skills in Japan.

## Methodology

This cross-sectional study was conducted twice, from January 18, 2021, to February 1, 2021, and from November 1, 2021, to November 14, 2021. We emailed questionnaires to all 4200 JSGOE members who were reachable via the Internet, and the responses were submitted anonymously using Google Forms. The questionnaire was designed by members of the JSGOE Technical Certification System for Gynecological Robotic Surgery task force. The primary endpoint was to determine how the members felt about developing a certified training facility and a technical certification system for robotic surgery. The secondary endpoint was to determine what type of system design would be considered important. This questionnaire study did not require ethical approval.

## Definition of an accredited training facility and technical certification system

### Certified training facilities

An accredited training facility for robotic surgery refers to a facility certified by the JSGOE as one in which the quality and safety of robotic surgery are assured. Although specific details have not yet been formulated, the goal is to ensure that safe surgery is performed through appropriate institutional management. The objectives of the JSGOE-accredited training facility in laparoscopy are as follows: to evaluate the skills and knowledge of endoscopic surgeons in the field of obstetrics and gynecology, to certify facilities where endoscopic surgery can be safely and smoothly performed, to promote the development and spread of endoscopic surgery in the field of obstetrics and gynecology in Japan, and to contribute to the maintenance of the health of the people.

### Technical certification system

 The robotic surgery technical certification system evaluates the skills of individuals. Although a specific evaluation system has not yet been established, the academic society properly evaluates surgical videos and certifies those whom they judge to have excellent skills. In Japan, a system of certified laparoscopic surgeons has been established in the field of gynecology, and there are currently 1090 certified laparoscopic surgeons in Japan. The objectives of the laparoscopic certification program are presented below:

Endoscopic surgery is a procedure performed in a closed space using delicate peripheral equipment that requires sufficient knowledge of the equipment and advanced techniques. The JSGOE technical certification system is designed to evaluate the skills and knowledge of endoscopic surgeons in the field of obstetrics and gynecology, certify those who have the skills to perform endoscopic surgery safely and smoothly and have the qualifications to serve as leaders in endoscopic surgery, promote the development and spread of endoscopic surgery in the field of obstetrics and gynecology in Japan, and contribute to the maintenance of the health of the people.

### Questionnaires

The questionnaire collected information on age, sex, prefecture, primary place of employment (university hospital, university-affiliated hospital, national hospital, city hospital, clinic, etc.), membership in the Japanese Society for Gynecologic Robotic Surgery (JSGRS), endoscopic technical certification by the JSGOE, gynecologic oncologist certification by the Japan Society of Gynecologic Oncology, total number of laparoscopic hysterectomy (TLH) procedures (0, < 10, 10–50, 50–100, and > 100), whether the facility has obtained the certification offered by da Vinci, the type of equipment installed in the da Vinci surgical system™ at the facility (Si, X, and Xi), number of robotic surgeries performed annually at their institution (0, < 10, 10–30, 30–50, and > 50), number of robotic surgeries they have performed (0, < 10, 10–30, 30–50, and > 50), how they are involved in robotic surgery (proctor, primary surgeon, assistant surgeon, and not involved), whether there is a need for a system of certified facilities for physician training, whether there is a need for a system for technically certifying physicians, and whether they would like to obtain a certification if such a system is established. If a technical certification system was to be established, the respondents were asked to choose from the following four options as to what type of system should be designed:

- Certification system for robotic surgery alone (with or without certification in laparoscopy, but experience in laparoscopic surgery is a requirement).
- Certification system for robotic surgery alone (those who have obtained certification in laparoscopic surgery can easily obtain this certification, and those who have not obtained certification in laparoscopic surgery are not required to have experience in laparoscopic surgery).
- Incorporation of robot-assisted surgery into the current certification system, considering it as a device used in endoscopic surgery.
- Other (the respondents could provide their own suggestions).

## Statistical analysis

We used the Chi-square test and Fisher’s exact test to investigate the significance of differences. Significance was set at *P* < 0.05. All statistical analyses were performed using GraphPad Prism 8.3 software (GraphPad Software, Inc., La Jolla, CA, USA).

## Results

**Fig. 1:**
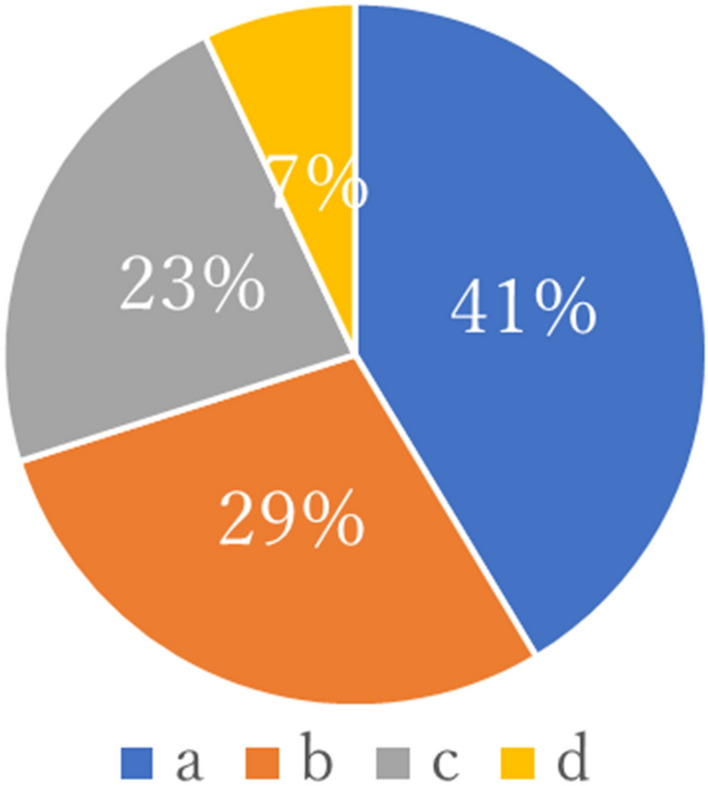
Design for the technical certification system, (**a**) Certification system for robotic surgery alone (with or without certification in laparoscopic surgery, but experience in laparoscopic surgery is a requirement). (**b**) Certification system for robotic surgery alone (those who have obtained certification in laparoscopic surgery can easily obtain this certification, and those who have not obtained certification in laparoscopic surgery are not required to have experience in laparoscopic surgery). (**c**) Incorporation of robot-assisted surgery into the current certification system, considering it as a device used in endoscopic surgery. (**d**) Other (the respondents could provide their own suggestions) Most respondents thought that the establishment of a certification system for robotic surgery alone (but with the requirement of experience in laparoscopic surgery) would be best, followed by a certain exemption for those who have obtained certification in laparoscopic techniques.

In total, 870 and 519 of the members who were contacted responded to the first and second questionnaires, respectively. Of the respondents to the first questionnaire, 31.8% (277/870) answered the second questionnaire. Details of the respondents’ attributes are shown in Table 1. Compared with the first survey, there was an increase in the percentage of women, JSGRS members, gynecologic oncologists, and those involved in robotic surgery in the second survey. Table 2 shows the number of TLH surgeries, robotic surgery experiences, and robotic operations at the facility. In the second survey, the number of robotic surgeries was higher than that in the first survey, and the experience of performing the surgeries also increased significantly. Tables 3 and 4 present the opinions regarding the certified facilities and technical certification system in each questionnaire. Overall, about half of the respondents believed that certified training facilities and a technical certification system are necessary, and the younger the respondent, the more important they felt it was. The breakdown by certification is presented in Tables 5 and 6. While gynecologic oncologists did not favor the technical certification system, a majority of those with experience of > 30 cases of robotic surgery felt the need for both accredited training facilities and a technical certification system (Table 5). The largest number of respondents thought that the best way to establish a certification system for robotic surgery was to establish this system alone (with the requirement of experience in laparoscopic surgery but with certain exemptions for those who have obtained certification in laparoscopic techniques; Fig. 1).

**Table 1:** Background characteristics of the respondents

		First survey (*n* = 870)	Second survey (*n* = 519)	*P* value
Sex	Male	603	192	< 0.01
	Female	267	327	
Age, years	20–29	15	2	< 0.01
	30–39	207	92	
	40–49	350	215	
	50–59	224	145	
	≥ 60	74	65	
Affiliations	University hospital	313	202	NS
	University-affiliated hospital	121	77	
	National hospital	3	1	
	Municipal hospital	395	215	
	Clinic	37	22	
	Others	1	2	
JSGRS member	Yes	236	226	< 0.01
	No	634	293	
Obtained JSGOE endoscopy certification	Yes	484	319	< 0.01
	No	386	200	
JSGOE Gynecologic oncology specialist qualification	Yes	320	234	< 0.01
	No	550	285	
Type of da Vinci Surgical System	Si	180	98	< 0.01
	X	79	58	
	Xi	383	271	
	SP	2	1	
	None	226	91	
Surgical system operating qualification	Surgeon console	360	297	< 0.01
	Assistant	62	28	
	None	448	194	
Involvement in robotic surgery	Proctor	34	33	< 0.01
	Surgeon	237	211	
	Assistant	119	62	
	None	480	213	

*JSGRS *Japanese Society for Gynecologic Robotic Surgery, *JSGOE* Japan Society of Gynecologic and Obstetric Endoscopy and Minimally Invasive Therapy, NS not significant

**Table 2:** Number of total laparoscopic hysterectomy and robotic surgery experiences and the number of robotic operations at the facility

		First survey (*n* = 870)	Second survey (*n* = 519)	*P* value
Number of total laparoscopic hysterectomies performed	0	52	26	NS
	1–9	69	42	
	10–50	184	104	
	51–100	151	88	
	> 100	414	259	
Number of robotic surgery cases per year	0–9	390	187	< 0.01
	10–29	213	113	
	30–49	125	78	
	≥ 50	142	141	
Number of robotic surgery experiences	0	537	232	< 0.01
	1–9	128	75	
	10–29	109	96	
	30–49	45	46	
	≥ 50	51	70	

*NS* not significant

**Table 3:** Results and breakdown of responses to each system at the time of the first survey

Age, years	Certified training facility			Technical certification system	
	Necessary	Unnecessary	Unknown	Necessary	Unnecessary
20–39 (*n* = 222)	123 (55.4%)	52 (23.4%)	47 (21.1%)	108 (48.6%)	114 (51.3%)
40–49 (*n* = 350)	174 (49.7%)	89 (25.4%)	87 (24.8%)	162 (46.2%)	188 (53.7%)
> 50 (*n* = 298)	141 (47.3%)	83 (27.8%)	74 (24.8%)	134 (44.9%)	164 (55.0%)
*P* value	NS			NS	

Data are presented as *n* (%), *NS* not significant

**Table 4:** Results and breakdown of responses to each system at the time of the second survey

Age, years	Certified training facility			Technical certification system		
	Necessary	Unnecessary	Unknown	Necessary	Unnecessary	Unknown
20–39 (*n* = 94)	56 (59.5%)	20 (21.2%)	18 (19.1%)	43 (45.7%)	37 (39.3%)	14 (14.8%)
40–49 (*n* = 216)	105 (48.6%)	68 (31.4%)	43 (19.9%)	79 (36.5%)	99 (45.8%)	38 (17.5%)
> 50 (*n* = 210)	123 (58.5%)	64 (30.4%)	23 (10.9%)	87 (41.4%)	89 (42.3%)	34 (16.1%)
*P* value	0.02			NS		

Data are presented as n (%), *NS* not significant

**Table 5:** Breakdown of opinions for technical certification system, breakdown of opinions for the certified training facility

	Necessary		Unnecessary		Unknown
	First	Second	First	Second	Second
	*n* = 404	*n* = 208	*n* = 466	*n* = 225	*n* = 86
Certified laparoscopic specialist	46.9	43.8	53	42.6	13.4
Board-certified in gynecologic oncology	43.7	34.1	56.2	49.5	16.2
Certified as a Da Vinci surgeon	47.2	41.1	52.7	45.9	13.9
Experienced robotic surgeon with > 30 cases	58.3	42.1	41.6	43.8	14
Mainly involved as proctors and surgeons	48.7	40.4	51.2	44.2	15.2

	Necessary		Unnecessary		Unknown	
	First	Second	First	Second	First	Second
	*n* = 438	*n* = 284	*n* = 224	*n* = 152	*n* = 208	*n* = 83
Certified laparoscopic specialist	50.0	57.0	23.3	30.4	26.7	12.5
Board-certified in gynecologic oncology	50.0	55.9	27.8	32.4	22.2	11.1
Certified as a Da Vinci surgeon	53.8	56.2	28.6	32.3	17.6	11.4
Experienced robotic surgeon with > 30 cases	66.6	59.3	18.7	28.4	14.7	12.0
Mainly involved as proctors and surgeons	56.0	56.1	26.1	32.3	17.6	11.4

Data are presented as percentages

## Discussion

This study examined how a technical certification system for robotic surgery, similar to that for laparoscopic surgery, should be designed, given the history of decreased incidence of complications due to the technical certification system for laparoscopic surgery. To our knowledge, this is the first study to conduct such a questionnaire survey, and the results may be important for the dissemination of robotic surgery, especially in developing countries. Half of the respondents deemed each system to be important. Thus, it is essential that each system is given importance as younger generations come to predominate in the field.

Robotic surgery is not yet widespread, or the topic is not of interest to many members. However, the number of robotic surgeries is clearly increasing (Table 2); thus, the demand for a system for robotic surgery may increase in the future. The JSGOE is conducting the same complication survey for robotic surgery that it conducted for laparoscopic surgery; therefore, it will be clearer in the future how many complications actually occur in robotic surgery compared with laparoscopic surgery.

In Japan, most robotic and laparoscopic surgeries are currently performed by experienced gynecologists aged > 40 years. In both surveys, the percentage of respondents who had experienced > 100 TLH cases was higher for those aged ≥ 40 years than for those in their 20 s and 30 s. Additionally, more than half of those aged ≥ 40 years reported having performed > 100 TLH cases. Thus, compared with open surgery, the generation that has begun performing robotic surgery is older, and young gynecologists have not yet had the opportunity to perform robotic surgery. However, according to our results, younger physicians are more likely to believe that accredited training facilities and technical certification systems are necessary. Although we did not formulate a question for this, the reason that they think a system is necessary even though they have not had the opportunity to perform laparoscopic surgery is probably because they have seen videos of senior doctors performing laparoscopic surgery and that they think about the presence of an element of instability rather than about performing laparoscopic surgery. Conversely, in the second survey, the percentage of respondents in their 50 s who had more opportunities to perform surgeries and who answered that the system was necessary was higher. The number of workshop participants was high, indicating interest in the technical certification system and certified training facilities; however, many participants expressed dissenting opinions. This may be because these participants did not fully understand the advantages of robotic surgery compared with those of laparoscopic surgery.

There are several training systems for learning robotic surgery in other countries, and they are sought after [11–16]. This is true in gynecology, urology, and surgery. A survey among the Brazilian College of Surgeons about the Associação Médica Brasileira (AMB—Brazilian Medical Association) statement on the new certification process for robotic surgery revealed that there was an overall agreement on the AMB statement among the Brazilian surgeons [17]. The authors concluded that the AMB statement on the new certification process seemed to be “a promising pathway to increase the participation of the medical entities into the robotic certification in Brazil” [17]. In another study, the authors from the Robotic Surgery Committee of the Brazilian College of Surgeons concluded that the formulation of robotic surgery certification norms could even encourage hospitals to adopt objective qualifications criteria for robotic surgery procedures[18]. However, currently, there is no surgical training system for robotic surgery in Japan. Thus, utilizing existing technical certification systems and certified training facilities in laparoscopic surgery for robotic surgery may be efficient. However, to ensure safe implementation, it may be important to initially build education through simulation of robotic surgery techniques.

Diverse curricula have emerged to train surgeons in robotic surgery [19]. Existing educational modalities are evolving to meet the rapidly growing demand for robotic surgeons. Future growth areas require establishing competency benchmarks for existing training tools, validating existing curricula, and determining how to translate skills acquired in simulation into performance in the operating room and patient outcomes. Many existing surgical training platforms are expanding beyond individual robotic skill training to procedure-specific team training. Understanding how to create an effective training experience for gynecologic surgical trainees and robotics teams warrants further research. If Japan develops a system that does not exist anywhere else, it is not enough to do so based on the opinions of obstetricians and gynecologists alone; public opinion is required. We plan to conduct a large-scale questionnaire survey of the Japanese public, particularly women and patients with gynecological diseases. Based on the results of that survey, we will decide on further directions.

This study has some limitations. First, only those who were involved in or interested in robotic surgery may have answered the questionnaire. This hypothesis is supported by the fact that the use of robotic surgery continues to be less common than laparoscopic surgery in Japan. The low answering rate in the 1st and 2nd questionnaires may have resulted from the limited number of JSGOE members being familiar with robotic surgery. Second, the application of robotic surgery remains limited in Japan. This can be partially attributed to the fact that social insurance coverage for gynecological surgeries began in 2018.

Herein, we found that there is no such system recognized by academic societies worldwide; thus, the construction of educational curricula using tools with functions, such as simulation-based surgical education and prediction of training effects of new modalities, may be important.

## Data Availability

All data generated or analyzed during this study are included in this published article.
